# Differential Scanning Fluorometry Signatures as Indicators of Enzyme Inhibitor Mode of Action: Case Study of Glutathione S-Transferase

**DOI:** 10.1371/journal.pone.0036219

**Published:** 2012-04-30

**Authors:** Wendy A. Lea, Anton Simeonov

**Affiliations:** NIH Chemical Genomics Center, National Human Genome Research Institute, National Institutes of Health, Bethesda, Maryland, United States of America; MRC National Institute for Medical Research, United Kingdom

## Abstract

Differential scanning fluorometry (DSF), also referred to as fluorescence thermal shift, is emerging as a convenient method to evaluate the stabilizing effect of small molecules on proteins of interest. However, its use in the mechanism of action studies has received far less attention. Herein, the ability of DSF to report on inhibitor mode of action was evaluated using glutathione S-transferase (GST) as a model enzyme that utilizes two distinct substrates and is known to be subject to a range of inhibition modes. Detailed investigation of the propensity of small molecule inhibitors to protect GST from thermal denaturation revealed that compounds with different inhibition modes displayed distinct thermal shift signatures when tested in the presence or absence of the enzyme's native co-substrate glutathione (GSH). Glutathione-competitive inhibitors produced dose-dependent thermal shift trendlines that converged at high compound concentrations. Inhibitors acting via the formation of glutathione conjugates induced a very pronounced stabilizing effect toward the protein only when GSH was present. Lastly, compounds known to act as noncompetitive inhibitors exhibited parallel concentration-dependent trends. Similar effects were observed with human GST isozymes A1-1 and M1-1. The results illustrate the potential of DSF as a tool to differentiate diverse classes of inhibitors based on simple analysis of co-substrate dependency of protein stabilization.

## Introduction

A range of biophysical techniques are used to evaluate direct binding between a ligand (most frequently, a small molecule) and a target protein, and these can be based on calorimetry, surface immobilization, separation, or direct spectroscopic methods [1]. A general method to evaluate compound-protein interaction is based on the ability of equilibrium binding ligand to perturb the protein stability upon application of a destabilizing factor, such as temperature, denaturing chemical, or proteolytic enzyme [1]. Although many techniques, such as NMR, MS or calorimetry, can monitor ligand-induced protein perturbation, their utility is often limited by complexity and requirements for high protein consumption [1], [2]. A method that overcomes some of these limitations is the fluorescence-based thermal shift assay, also known as differential scanning fluorometry (DSF). In DSF, an environmentally sensitive fluorescence dye whose quantum yield increases upon binding to hydrophobic protein regions is applied to monitor protein conformational stability upon thermal denaturation [3], [4]. By coupling ligand binding to protein unfolding, protein Gibbs free energy of unfolding is increased, usually resulting in an increase in protein melting temperature, T_m_, which in turn can be used as an indicator of a direct protein binder.

Execution of DSF does not involve any modification of the protein target or separation steps, and it does not require any prior knowledge of (but may assist to elicit) protein function [2], [3], [5], [6], [7], [8], [9]. DSF has been used to assist with refining protein crystallization conditions and has been reported to allow the determination of ligand-binding affinity [10], [11] or binding stoichiometry [11]. T_m_ shift has been shown to correlate well with enzyme inhibition data or binding affinities derived from other methods [12], [13], [14]. Two recent studies exemplify the use of DSF to conduct more complex studies, such as the probing of co-factor dependencies of inhibitor binding to 15-hydroxyprostaglandin dehydrogenase [15] and the demonstration of an enhanced stabilization effect on firefly luciferase reporter through reaction between the small molecule agent PTC124 and ATP [16]. Despite these advances, the majority of uses of DSF have been confined to prioritization of ligands for X-ray crystallography, as well as limited medium-throughput screening, typically executed at a single compound concentration.

We wished to further leverage the information provided by DSF and, in particular, to explore its ability to distinguish inhibitors acting by different mechanisms. To this end, we used glutathione S-transferase (GST) as a model enzyme for a relatively complex reaction involving two distinct substrates and known for being inhibited through a range of mechanisms. GSTs contribute to the phase II biotransformation of xenobiotics in a variety of organisms, with members of the family being involved in both the metabolism and transportation of potentially toxic ligands. These functions are accomplished either through the catalytic conjugation of a variety of electrophiles with glutathione (γ-Glu-Cys-Gly, GSH) by GST or ligandin binding ability of GST with a range of lipophilic chemicals [17]. Based on sequence similarity and substrate specificity, human cytosolic GSTs are generally divided into 5 classes, designated as alpha (A class), mu (M class), pi (P class), theta (T class) and kappa (K class) [18]. GSTs have also been detected in a range of pathogenic helminths, such as *Schistosoma* worms, and in the malarial parasite *Plasmodium falciparum*. GSTs have been extensively studied for their association with cancer. Genetic polymorphisms in human GSTs have been linked with oxidative DNA damage and subsequently an increased risk of cancer susceptibility [19], while schistosomal GSTs have been considered as potential components in vaccines [20] and as targets for schistosomiasis drug therapy [21]. *Schistosoma japonicum* glutathione S-transferase (EC 2.5.1.18) (*Sj*GST) is also a commonly used fusion tag in recombinant protein production [22].

A number of apo-protein structures of human [23], [24] and *Schistosoma japonicum* [21], [25] have been published, as well as structures of protein-ligand complexes [26], [27], [28], [29]. GSTs are either homo- or heterodimers, with an active site in each monomer. Each subunit contains two domains, an N-terminal α/β domain and a C-terminal α-helical domain. A highly selective glutathione-binding site (G site) is located in the N-terminal domain and a larger hydrophobic substrate-binding site (H site) is located in the C-terminal domain. Although the former is more conserved across different classes than the latter [30], [31], [32], the two adjacent sites work together to promote GSH conjugation with electrophilic substrates. Additionally, a non-substrate ligand transport site (L site), suggested to be not completely hydrophobic, has been identified for *Sj*GST [21] and a human π class GST [33], and a large variability in its location among different species has been observed.

GST inhibitors have been demonstrated as chemosensitisers to potentiate anticancer agents [31]. In addition, the discovery of GSTs' regulation in signal transduction pathways through specific protein-protein interactions (PPIs), such as the interruption of the cJun/MAPK pathway by GSTP1-1 [34], [35] and the formation of inhibitory complexes with the apoptotic stress kinase ASK1 by GSTM1-1 [36], provides a rationale in the design of GST inhibitors to potentially disrupt PPIs. Several types of GST inhibitors are known and, based on their binding site and inhibition mechanism, they are generally categorized into the following classes. One type is represented by GSH analogs and mimetics, which compete with both GSH and hydrophobic substrates by occupying both the G-site and the H-site [37]. The second class is comprised of certain electrophilic substrates, which bind in the hydrophobic region of the H-site and form tight complexes with GST through the formation of adducts with the GSH co-substrate [37], [38]. In addition, a number of compounds are found to be noncompetitive inhibitors with respect to both GSH and electrophilic substrate and they are believed to partially occupy either the H-site or the intersubunit cleft of the GST dimer [37], [38]. As the nature of the binding site for this type of inhibitors is less defined, this type of inhibitors is also referred to as ligandin type inhibitors and their binding site has been named the ligandin site [17].

The availability of multiple types of GST inhibitors presents this enzyme class as an attractive model to dissect ligand-dependent protein stabilization effects, and to further evaluate the ability of DSF as a tool to provide insight on compound mechanism of action. Herein, different classes of GST inhibitors were selected, and their effects on thermal stability of *Sj*GST and the human GST A1 and M1 were examined. Protein thermal stability was measured in the presence of multi-point dilution series of inhibitors; further, DSF signatures obtained in the absence and presence of GSH were evaluated for indication of compound mechanism of action.

## Materials and Methods

### General reagents

HEPES buffer (pH 7.5) was purchased from Teknova. Tween-20 and glutathione (GSH) were obtained from Sigma-Aldrich (St Louis, MO). Dimethylsulfoxide (DMSO, certified ACS grade) was obtained from Fisher, Inc. All compounds were formulated as 10 mM DMSO stock solutions. The buffer used for both the enzymatic and the thermal shift assay was 50 mM HEPES, pH 7.5, except that Tween-20 was present in the enzymatic assay at a final concentration of 0.01%. The fluorescent dye used in the thermal shift experiments, SYPRO Orange, was obtained from Life Technologies (Carlsbad, CA) as a 5000× stock concentration (molar concentration of the stock is not provided by the vendor), and was diluted in the assay buffer to a final concentration of 5×.

### GST enzymes, substrates, and inhibitors

*Sj*26GST was purchased from GenScript (Piscataway, NJ). Human GST isozymes, hGST A1-1 and hGST M1-1, were procured from Oxford Biomedical Research (Oxford, MI). The masked proluciferin substrate PBI 1155 [39] and the inhibitors NBD-8-OH [40], NBD-GS [41], and Bis-(NB-GS) [38] were purchased from Promega, Inc. (Madison, WI). S-methyl GSH, S-butyl GSH, S-hexyl GSH, S-octyl GSH, ethacrynic acid (EA), quercetin, myricetin and tannic acid were obtained from Sigma (St Louis, MO).

### *Sj*GST enzymatic assays

Compound inhibitory activities were evaluated using a previously reported GST enzymatic assay [39]. Three µl of reagents (5 nM final concentration of *Sj*GST (or buffer serving as a no-enzyme control) plus 100 µM final concentration of GSH) were dispensed into 1536-well Greiner white solid-bottom assay plates. Compounds (23 nL) were transferred via Kalypsys pintool equipped with 1,536-pin array [42]. The plates were incubated at room temperature for 15 min before the addition of 20 µM substrate PBI 1155 to initiate the reaction. The plates were centrifuged at 1000 rpm for 15 s and incubated at room temperature for 40 min before the addition 4 µl luciferin detection reagent followed by another 15-s centrifugation at 1000 rpm and 15-min incubation. The plates were then read on ViewLux high-throughput CCD imager (PerkinElmer, Waltham, MA) with a clear emission filter and under standard luminescence settings. Percent inhibition was calculated based on the enzyme-containing and no-enzyme controls using Excel and GraphPad Prism 4.

### Thermal shift assays

Compounds were diluted (1∶2, 7-points) in DMSO row-wise down to 10 µM in a 96-well polypropylene round bottom mother plate, with DMSO alone in the first column of each plate. After distributing 49 µl *Sj*GST (1 µM final concentration) and SYPRO Orange (5× final concentration) mixture into the wells of a 96-well thin wall PCR plate (Bio-Rad, Hercules, CA), compounds (1 µl) were transferred from the mother plate to the PCR plate with the final concentrations ranging from 0.2 to 200 µM; DMSO was included as a vehicle control at a final concentration of 2% (vol/vol), a suggested value for thermal shift experiment [7]; the inclusion of 2% DMSO resulted in only a marginal decrease in the *Sj*GST T_m_ of 0.95+0.07°C (n = 2) (data not shown). The PCR plates were centrifuged at 1000 rpm for 10 s to ensure good mixing, and sealed with Optical-Quality Sealing Tape (Bio-Rad). The plates were subsequently heated approximately 2 min after sample mixing, on an iQ5 thermal cycler at intervals of 1°C from 20 to 95°C, with a ramping rate of 6°C min^−1^. The set-up of the filter configurations was customized to accommodate the optimal excitation and emission wavelengths for SYPRO Orange (Ex: 490/Em: 575 nm). The mid temperature of the protein thermal melt profiles, T_m_, was determined using EXCEL-based custom calculation software available at (ftp://ftp.sgc.ox.ac.uk/pub/biophysics), and fitting of the data to the Boltzmann equation was performed using GraphPad Prism 4. The differences in T_m_ between vehicle control and compound-containing samples were calculated as thermal shift.

## Results

### Inhibitors selected for the study

**Figure 1:**
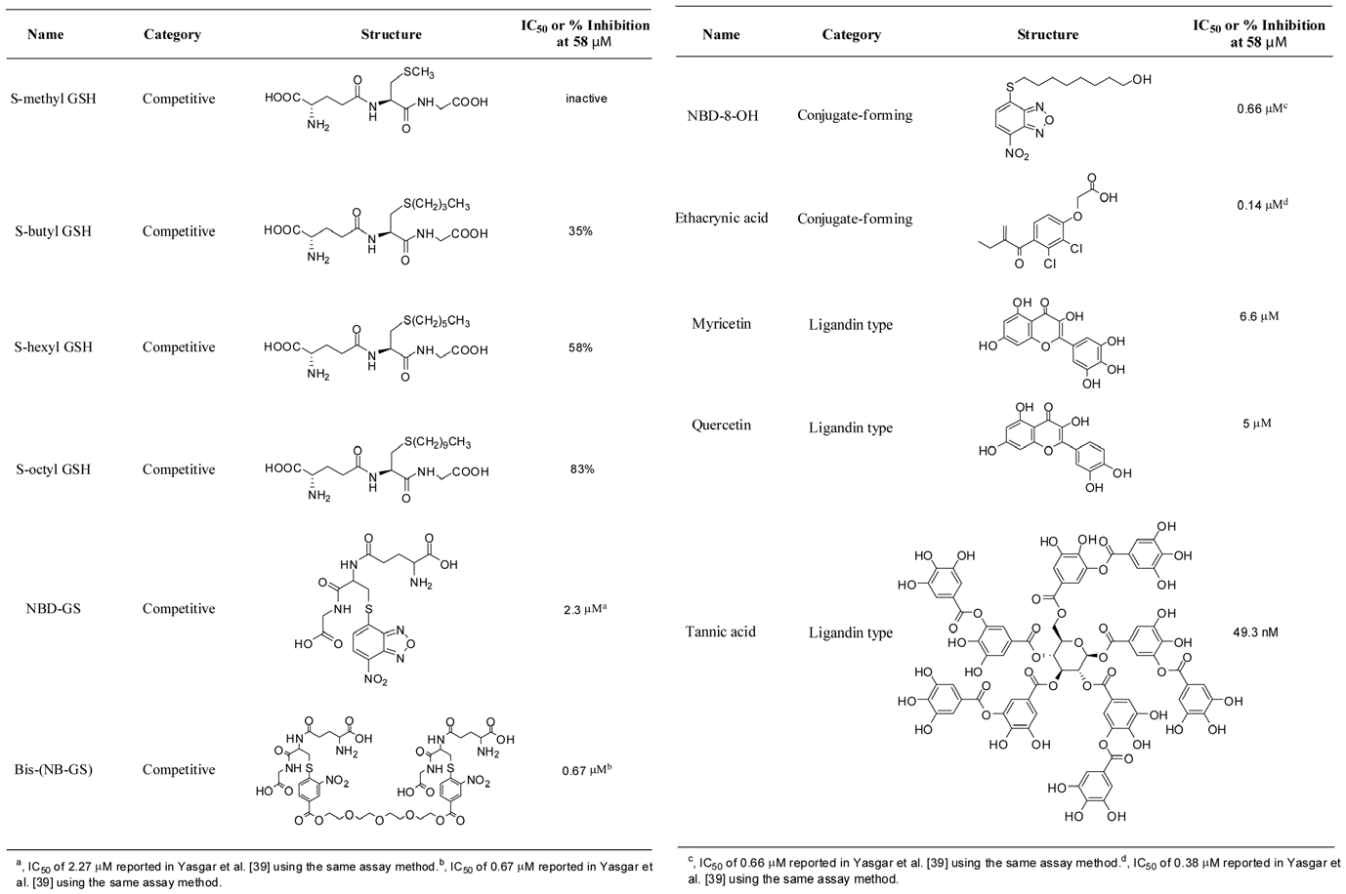
GST inhibitors tested in this study and their inhibitory activity against *Sj*GST.

Three categories of GST inhibitors were chosen for this study (Fig. 1). GSH analogs and mimetics (alkyl-GSHs, NBD-GS, and Bis-(NB-GS)) represented GSH-competitive inhibitors [38], [43]. EA and NBD-8-OH represented the category of compounds known to form conjugates with GSH [30]. Lastly, quercetin, myricetin, and tannic acid belonged to the ligandin-type inhibitor category [30]. Although a range of inhibitory potencies have been reported for these compounds in multiple studies spanning decades, we wished to profile all of them using a recently developed sensitive luminescence-based enzymatic assay [39]. The IC_50_ values derived from the luciferase-coupled enzymatic assay are shown in Fig. 1 along with the compounds' structures.

Due to the limited solubility of S-alkyl GSHs, a lack of inhibitory saturation was observed at top concentrations tested, making it impossible to derive IC_50_ values. Thus, the percent inhibition caused by these molecules at a fixed concentration was used to represent and compare their activity against *Sj*GST. The S-alkyl glutathione derivatives displayed alkyl-chain dependent enzyme inhibitory activity with greater inhibition corresponding to derivatives with longer alkyl chain, in agreement with previously noted trends [44]: at 58 µM, 83% inhibition was obtained for S-octyl GSH, 58% for S-hexyl GSH, and 35% for S-butyl GSH; S-methyl GSH did not show detectable inhibition when tested at concentrations up to 200 µM. Between the two GSH analogs, the bivalent compound Bis-(NB-GS), exhibited an order of magnitude lower IC_50_ than NBD-GS. The third NBD compound, NBD-8-OH, and EA both produced submicromolar IC_50_s. Among the rest of the compounds, myricetin, and quercetin exhibited single digit micromolar IC_50_ values, while tannic acid was the most potent inhibitor tested, yielding a double digit nanomolar IC_50_.

### *Sj*GST thermal denaturation profiles

**Figure 2:**
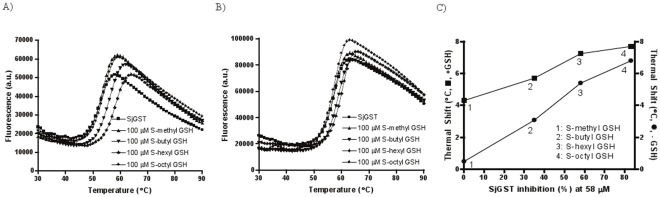
Thermal denaturation curves for *Sj*GST alone or with 100 µM S-alkyl GSH in the absence (A) and presence (B) of 2 mM GSH; (C) correlation between thermal shift at 100 µM alkyl-GSH and degree of inhibition against *Sj*GST. Thermal shifts, where applicable, represent the differences in T_m_ between vehicle control and compound-containing samples, respectively.

Thermal denaturation profiles of *Sj*GST were recorded following the fluorescence change of the environmentally sensitive dye SYPRO Orange (Fig. 2 A and B). The profiles contained a single transition and an asymmetric peak, with a maximum fluorescence intensity achieved at approximately 60°C. The decrease in fluorescence on the right-hand side of the peak is typically attributed to aggregation and precipitation of the denatured protein, as well as to the natural decrease in quantum yield of the fluorescent dye at higher temperatures. A T_m_ of 53.0±0.02°C (n = 2) was obtained with high reproducibility for the apo protein (see raw-fluorescence plots of duplicate determination of the melting profile for SjGST in Supplemental Figure S1); this value was similar to those obtained by measuring *Sj*GST thermal denaturation through its residual enzymatic activity after heating (T_m_∼51°C) [22] and by differential scanning calorimetry (T_m_: 55.15–58.95°C at pH 7.5 using different scanning rates) [45]. Using differential scanning calorimetry, Quesada-Soriano *et al*. also found that a single melt transition was consistently produced at various *Sj*GST concentrations. The clear monophasic thermal transition of *Sj*GST obtained under the DSF conditions here permitted subsequent tests where compounds of interest were included.

### *Sj*GST DSF profiles with GSH mimetics

**Figure 3:**
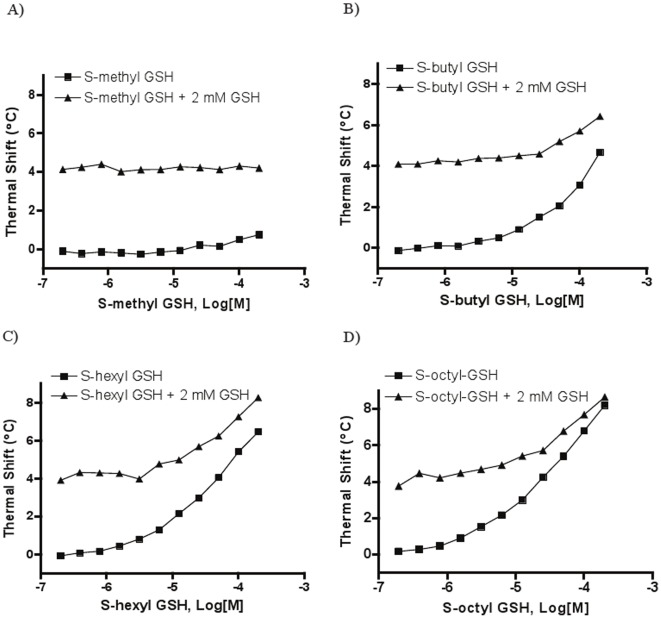
Thermal shift concentration-response curves for S-alkyl GSH using *Sj*GST: A) S-methyl GSH, B) S-butyl GSH, C) S-hexyl GSH, D) S-octyl GSH. Thermal shifts represent the differences in T_m_ between vehicle control and compound-containing samples, respectively.

*Sj*GST thermal stability was tested in the presence of a series of S-alkylglutathione derivatives (representative melting curves shown in Fig. 2A). Ligand-induced protein stabilization effect was apparent for all compounds tested, and protein T_m_ increased with increasing concentrations of ligand (Fig. 3, square symbols in all panels). The corresponding magnitudes of protein stabilization were different among different S-alkylglutathione derivatives. The rank order of thermal stabilization observed was: S-octyl GSH>S-hexyl GSH>S-butyl GSH>S-methyl GSH. Overall, there was a good correlation between the thermal shifts produced by the S-alkyl GSHs and their inhibitory effects on *Sj*GST (Fig. 2C).

When the S-alkylglutathiones were tested in the presence of 2 mM GSH (representative melting curves shown in Fig. 2B), a concentration above the reported Km value chosen to ensure adequate occupancy of the corresponding binding site, a ∼4°C enhancement in the thermal shift baseline was observed at low compound concentrations, due to the stabilizing effect afforded by GSH, but that enhancement gradually decreased as the compound concentration increased, leading to the two curves converging (Fig. 3). The converging effect became stronger as the alkyl chain increased: S-methyl glutathione itself did not produce significant thermal shift (<2°C) and the thermal shift differences between the GSH-absent and GSH-present curves remained approximately 4°C, while the difference in thermal shifts at the top compound concentrations was compressed to 1.8°C for both S-butyl and S-hexyl glutathione, and further to 0.45°C for S-octyl glutathione.

**Figure 4:**
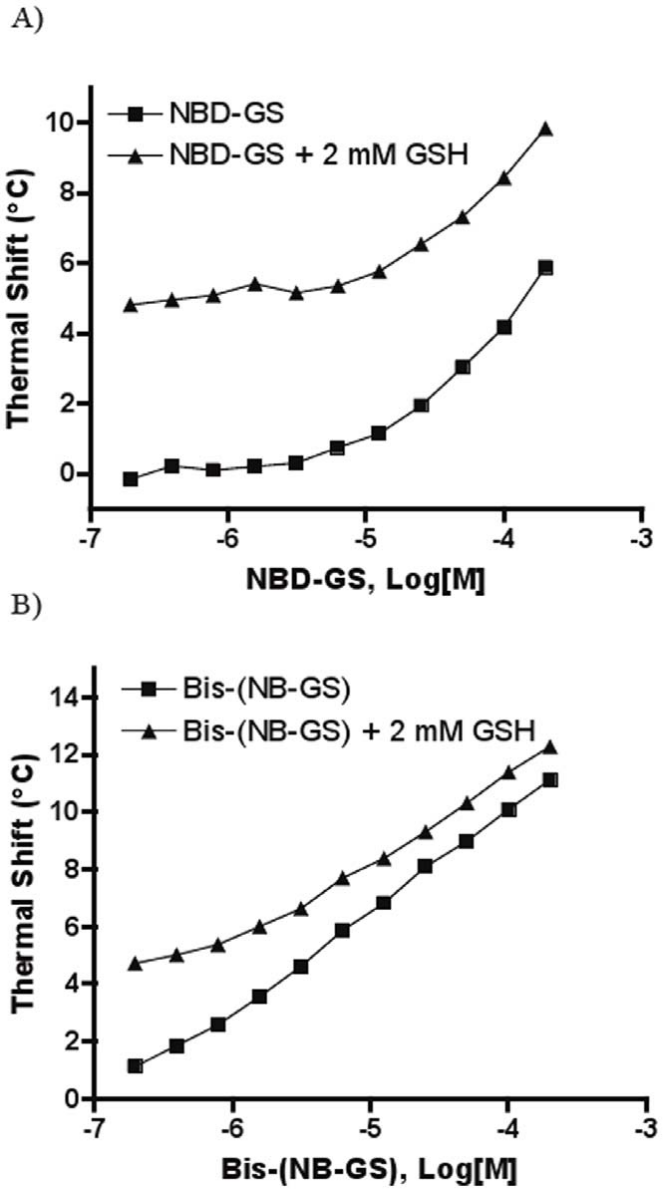
Thermal shift concentration-response curves using *Sj*GST in the absence and presence of 2 mM GSH for A) NBD-GS and B) bis-(NB-GS). Thermal shifts represent the differences in T_m_ between vehicle control and compound-containing samples, respectively.

Additional GSH analogs tested were Bis-(NB-GS) and NBD-GS. Bis-(NB-GS) induced a very large thermal stabilization, generating about twice the thermal shift produced by its monovalent version NBD-GS (11.1°C vs. 5.9°C at 200 µM, Fig. 4), in unison with the over 3-fold greater inhibitory potency of Bis-(NB-GS) versus NBD-GS (0.67 µM versus 2.3 µM, Fig. 1). Both Bis-(NB-GS) and NBD-GS displayed similar thermal shift profiles to those observed for the S-alkylglutathiones: the thermal shift enhancement produced in the presence of constant 2 mM GSH became more compressed as the inhibitor concentration increased. The converging trend between the thermal shift profiles obtained in the presence and absence of GSH was more pronounced for Bis-(NB-GS) than for NBD-GS: for Bis-(NB-GS), the difference between the T_m_ under GSH-present and GSH-absent condition decreased from the initial 4.8°C at concentration zero to 1.2°C at 200 µM inhibitor, while the corresponding value for NBD-GS only dropped from the initial 4.8°C to 4°C.

### *Sj*GST DSF profiles with GSH conjugate-forming inhibitors

**Figure 5:**
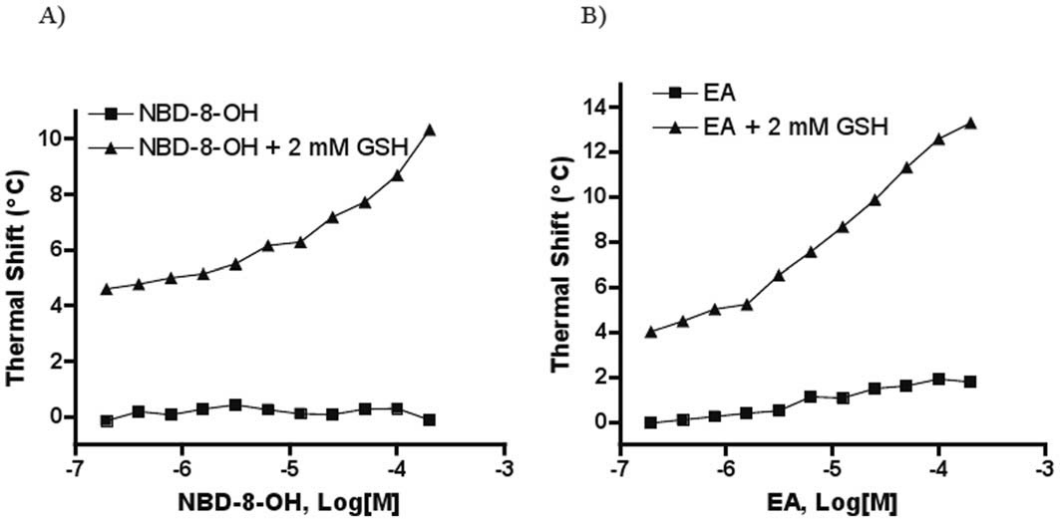
Thermal shift concentration response curves using *Sj*GST in the absence and presence of GSH for the conjugate-formers NBD-OH (A) and ethacrynic acid (EA, panel B). Thermal shifts represent the differences in T_m_ between vehicle control and compound-containing samples, respectively.

NBD-8-OH and ethacrynic acid (EA) produced similar thermal shift profiles (Fig. 5): in the absence of GSH, while the former failed to display any significant thermal stabilization effect and the latter elicited only a 1.8°C thermal shift at the top concentration, thermal stabilization effects induced by these two inhibitors became dramatically enhanced when 2 mM GSH was included in the test. Specifically, further enhancements of 10.5°C and 11.5°C were observed at 200 µM NBD-8-OH and EA, respectively, when GSH was present. It thus appeared that the strong thermal stabilization of GST by these inhibitors was strictly dependent on the presence of the glutathione co-substrate.

### *Sj*GST DSF profiles with ligandin type inhibitors

**Figure 6:**
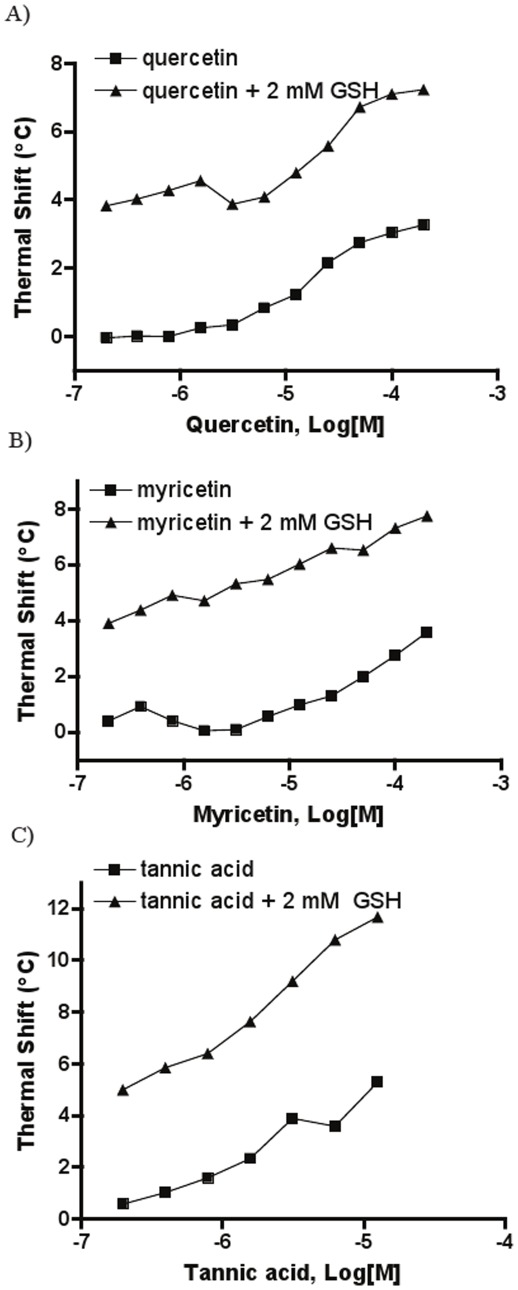
Thermal shift concentration-response curves using *Sj*GST in the absence and presence of 2 mM GSH for A) quercetin, B) myricetin and C) tannic acid. Thermal shifts represent the differences in T_m_ between vehicle control and compound-containing samples, respectively.

In the presence of GSH, quercetin, myricetin and tannic acid produced profiles parallel to those obtained in its absence. Both quercetin and myricetin increased the protein T_m_ by over 3°C at 200 µM without GSH, with the corresponding GSH-present curves simply upward-shifted by approximately 4°C across all concentrations (Fig. 6 A/B). Tannic acid, which could be tested only up to 12.5 µM due to interference with the DSF signal, also produced parallel trends separated by approximately 4.5°C (Fig. 6C). Thus, the stabilization afforded by the ligandin type inhibitors tested here appeared to be independent from the corresponding effect of GSH.

### DSF signatures of human GST A1 and M1

**Figure 7:**
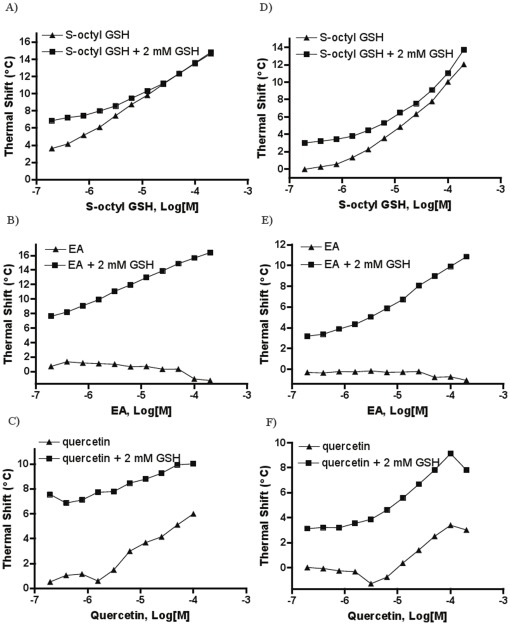
Thermal shift concentration-response curves using hGSTA1-1 in the absence and presence of 2 mM GSH for A) S-octyl GSH, B) ethacrynic acid (EA), C) quercetin, and using hGSTM1-1 for D) S-octyl GSH, E) EA, and F) quercetin. Thermal shifts represent the differences in T_m_ between vehicle control and compound-containing samples, respectively.

The study was subsequently extended to human GST isozymes by testing one representative compound from each category against hGST A1 and M1 (hGST P1 was not pursued due to the protein's lack of clear melt transition, data not shown). Thermal shift responses and glutathione-dependency trends of the inhibitors tested against hGST A1 and hGST M1 were similar to those obtained with *Sj*GST, that is, a converging trend for S-octyl GSH (Fig. 7A/D), a diverging trend for ethacrynic acid (Fig. 7B/E), and a parallel trend for quercetin (Fig. 7C/F).

## Discussion

**Figure 8:**
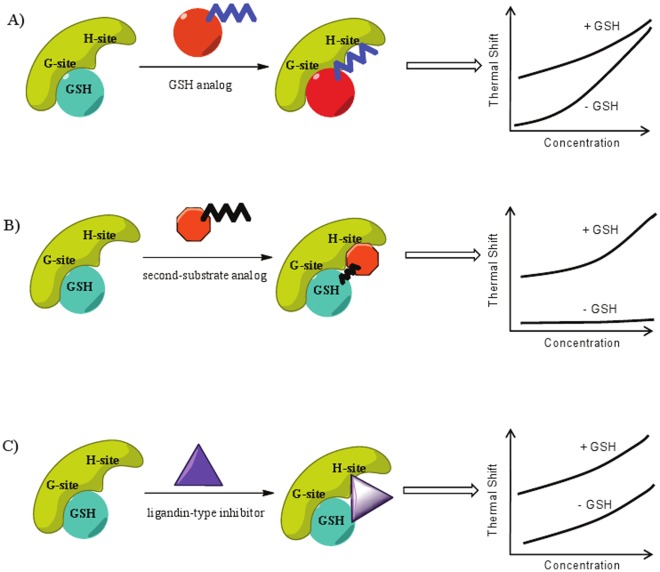
Schematic illustration of different inhibition mechanisms and the associated DSF signatures: A) GSH-competitive inhibition; B) conjugate-formation; C) ligandin-type.

The goal of this study was to apply GST as a model target in order to investigate whether ligands' mode of inhibition could be discerned through DSF signatures. To this end, we examined the thermal stability changes of *Sj*GST, hGST A1 and hGST M1 in the presence of three classes of inhibitors. By testing each inhibitor in concentration-response format and by comparing the compound-induced thermal stabilization effects in the absence and presence of GST's physiological substrate GSH, we derived thermal shift profiles for each compound studied and found that GST inhibitors from the same class showed similar DSF signatures, implying a common mechanism of action for these compounds. In turn, the three different classes of inhibitors produced markedly different co-substrate dependency signatures, consistent with their modes of action; the signatures derived in the present study are presented in schematic form in Fig. 8.

### GST interaction with GSH analogs

The compression phenomena observed for the S-alkylglutathiones in the presence of GSH are consistent with these compounds acting as competitive inhibitors of *Sj*GST with respect to GSH (Fig. 8A), as previously reported [43]; in addition, the magnitudes of thermal stabilization observed here correlated well with not only the S-alkylglutathiones' inhibitory activity against *Sj*GST measured in this study but also with their binding affinities against *Sj*GST reported by Ortiz-Salmerón *et al*. [43]. The polar G-site is conserved between parasitic and mammalian enzymes to sequester GSH or the GSH moiety from GSH-analogs through similar but specific hydrogen bonding interactions [27], [46]. The H-site has been found to vary at the sequence and structural level [27], and it has been suggested that the alkyl side chains make non-specific apolar contacts within the H-site, rendering additional binding energy and subsequently tighter binding [46]. Thus, our observation that a significantly higher degree of thermal shift was achieved for compounds with longer alkyl chains is consistent with the previous conclusions that increase in the length of the alkyl chain results in a more hydrophobic environment and thus a higher binding affinity contributed by the increased binding site complementarity [47].

NBD-GS and Bis-(NB-GS) are reported competitive inhibitors with respect to common GST substrates, such as GSH and CDNB [38] and in our GSH dependency testing of GST thermal stabilization both produced the same convergent trendlines. The presence of the thioether group was suggested to be essential for tight binding [40], in line with the single digit micromolar or submicromolar potencies displayed by these compounds. Bis-(NB-GS) is a symmetrical bivalent inhibitor of *Sj*GST, designed to occupy both active sites of the dimeric enzyme simultaneously. As GSTs have one active site in each monomer, the concept of “multivalency" is a strategy to design compounds with increased contacts with the protein's active sites simultaneously, which could potentially increase their binding affinity and selectivity [30], [31]. The approach has been validated using existing bivalent ligands, such as GS-CDNB conjugate and the large symmetrical dye Cibacron Blue [38]. A thermodynamics study by Lyon *et al*. attributed improved bivalent compound binding affinities to GST to their significantly more favorable binding enthalpy [38]. This is in agreement with the large protein stabilization effect observed with Bis-(NB-GS) in our study, as the magnitude of thermal shift is affected by several factors: for a given protein at a fixed concentration, the ligand concentration and its binding affinity, along with enthalpy and heat capacity of ligand binding, determine the extent of thermal shift [5], [11].

### GST interaction with GSH conjugate-forming inhibitors

Several classes of GSTs have been reported to be sensitive to product inhibition achieved through the formation of covalent GSH conjugate [48] (Fig. 8B). In contrast to NBD-GS and Bis-(NB-GS), NBD-8-OH does not contain a GSH moiety, but is designed to be a suicide inhibitor for GSTs due to its ability to form a GSH conjugate during the reaction [40]. This notion was supported by the significantly enhanced thermal stabilization effect induced by NBD-8-OH when GSH was present (Fig. 5). Through spectrophotometric and fluorometric analyses using an analog of NBD-8-OH, NBDHEX (6-methylene substituent in the S-side chain instead of the 8 methylenes contained within NBD-8-OH), Ricci *et al*. found that the inhibitor bound to the H-site and that the formation of GSH conjugate with the concomitant release of 6-mercapto-1-hexanol was greatly facilitated in the presence of the enzyme [40]. The strong association constants for the NBD-GS adduct determined for several GSTs in that study are hereby supported by the observation of a large thermal stabilization produced by NBD-8-OH/GSH and the very close resemblance of the thermal shift profiles between NBD-8-OH/GSH and NBD-GS/GSH.

The other model inhibitor used here, EA, is a potent diuretic drug and an inhibitor of multiple GSTs. Its conjugate with GSH, EA-GSH, suggested to be formed by Michael addition, is also a strong GST inhibitor [27], [28], [49]. Co-crystal structures of EA-GSH conjugate in complex with human α and π GST indicate the requirement of bound glutathione for EA to dock into the H site in a productive binding mode, as EA itself was found to bind in a less optimal mode to the H site (non-productive mode) with the G site occupied by solvent molecules [23], [28]. The position in which EA bound in the H site of the enzyme was also found to be similar to the hexyl moiety of the S-hexyl-GSH complex crystal structure [28]. Thus, the presence of GSH in the GST active site has been hypothesized to serve as a molecular recognition element necessary for EA to efficiently interact with GSTs [23]. In concert with these analyses, we observed a minimal thermal stabilization effect exerted by EA alone while a dramatically higher stabilization was obtained at the same EA concentration in the presence of GSH.

### GST interaction with ligandin-type inhibitors

Another type of GST inhibitors have been referred to as the ligandin type inhibitors, represented by hydrophobic planar aromatic compounds with anionic functional groups, such as porphyrins, polyphenols or tocopherols [30]. The exact binding site for ligandin type inhibitors remains to be fully characterized, but there has been speculation that their binding site (referred to as L-site) may be of a degenerate nature. Crystallographic studies by Oakley *et al*. suggested that the L-site was located in the H-site for hGST P1 [33] while McTigue *et al*. provided evidence that the L-site was located along the dimer interface in *Sj*GST and was the binding site for the anti-schistosomiasis agent praziquantel [21]. Findings from additional studies led to the notion that there might be an expansive ligandin site that spanned the intersubunit cleft and the H-site [30]. Multiple binding modes for ligandin-type inhibitors may exist, as extensive members of ligandin-type inhibitors, such as plant polyphenols and tocopherols, have been shown to be competitive towards hydrophobic substrate or involved in active site covalent modification [50] and will likely be different for the different chemical structural classes. The inhibition mechanisms of these compounds await further investigation.

Herein, we performed analyses of 3 ligandin type inhibitors, quercetin, myricetin and tannic acid. The parallel concentration-response thermal shift curves observed by us for these compounds in the presence and absence of GSH are indicative of stabilizing effects exerted by the inhibitors being independent of those caused by GSH and thus appear to support the mechanism where these compounds inhibit the enzyme by docking into the H site without significant interference with the G-site, that is, by being non-competitive with respect to GSH (Fig. 8C). Using GST isolated from rat livers, Merlos *et al*. showed that several flavonoids, including quercetin, behaved as a non-competitive inhibitor versus both GSH and CDNB [51].

The present study is, to our knowledge, the first to interrogate compound mechanism of inhibition for GST enzymes through fluorescence-based thermal shift assays. Based on the relative shapes of the thermal shift profiles produced with and without the native co-substrate GSH, an indication of compound inhibition mechanism is provided. Converging, diverging and parallel DSF signatures were linked to competitive, conjugate formation (product inhibition), and ligandin-type inhibition. Although electrophile binding site and substrate binding mode have been characterized for *Sj*GST and human isozymes using other methods, such as fluorescence spectroscopy [52], ITC [43], [52], crystallography and molecular docking [27], our study demonstrates that DSF can provide complementary information on protein-ligand binding pathways using very simple experimental setup which allows the rapid profiling of multiple inhibitors. The application of DSF as a tool to probe enzyme-ligand interaction mechanisms can be expanded to a wide range of protein classes and is expected to be particularly useful in situations where detailed enzymological and spectroscopic studies are difficult to implement.

## Supporting Information

Figure S1 **Reproducibility of thermal denaturation profiles.**
(DOC)
