# Supplementary material for: Differential Scanning Fluorometry Signatures as Indicators of Enzyme Inhibitor Mode of Action: Case Study of Glutathione S-Transferase
Source: PLoS One. 2012 Apr 30;7(4):e36219. doi: 10.1371/journal.pone.0036219 (PMC3340335; doi:10.1371/journal.pone.0036219)
Supplement: Figure S1 — Reproducibility of thermal denaturation profiles. (DOC) [file pone.0036219.s001.doc]

**Supplemental Figure S1. Reproducibility of thermal denaturation profiles.** Shown is the duplicate determination of the melting profile for *Sj*GST resulting in almost perfectly overlapping raw-fluorescence profiles and individual T_m_ values that differed by only 0.02 °C, well within the accuracy limit of the instrument.
